# Distributed Non-Fragile State Estimation for Uncertain Nonlinear Systems of Sensor Networks Subject to Sensor Nonlinearities

**DOI:** 10.3390/s25071962

**Published:** 2025-03-21

**Authors:** Shihui Tian, Ke Xu, Fengshan Huang

**Affiliations:** 1 Collaborative Innovation Center of Steel Technology, University of Science and Technology Beijing, Xueyuan Road 30, Haidian District, Beijing 100083, China; shtian@xs.ustb.edu.cn; 2 College of Mechanical Engineering, Hebei University of Science and Technology, Shijiazhuang 050018, China

**Keywords:** distributed state estimation, sensor network, sensor nonlinearities, non-fragile control

## Abstract

This paper studies the distributed state estimation issue of nonlinear dynamical systems with parameter uncertainties based on sensor networks under the non-fragile control framework. Moreover, all the sensors are in a fully distributed framework with information exchanges to reduce the communication and computation resources. In particular, the sensor nonlinearities in the sensor network and state estimation gain fluctuations are taken into account for more general applicability. With the help of the Lyapunov–Krasovskii approach, sufficient convex optimization criteria can be given so that the passivity performance of its resultant state estimation error system can be guaranteed. The optimized non-fragile state estimation gains can be further determined on the basis of solving the convex optimization. The advantages and usefulness of our developed results are finally demonstrated by two illustrative examples.

## 1. Introduction

With the substantial progress of advanced sensor technologies and computer networks, various ever-increasing applications of sensor networks across a wide range have been investigated and implemented such as environment monitoring [1], intelligent grids [2], manufacturing networks [3], and so on [4,5]. Under this context of sensor networks, a fundamental yet important issue is to estimate the states of certain target systems well. Generally speaking, a typical sensor network consists of an array of senor nodes with measurement sensing, computation, and information exchange capabilities [6,7]. To date, instead of a traditional sensor that works alone, the deployment of sensor networks that can work collectively can always simplify sensor node design involved with more robustness, more applicability, and lower cost [8,9]. The key idea behind sensor networks coupled over the monitored regions has been to communicate the local information among the neighboring sensor nodes via wired or wireless networks. On the other hand, there has long been ongoing research investigations with practical applications in the communication of information in sensor networks. It can be found that distributed sensor networks can further achieve more flexibility and higher reliability [10,11]. More specifically, for distributed sensor network designs, each sensor node in the group could generate the local signals from both sensor nodes and their neighboring nodes in accordance with essential information exchange topologies. While these merits and significant advantages can be enjoyed, they can also render additional design difficulties and challenges for the distributed framework. Fortunately, many remarkable related research results have been presented in the literature and the references therein [12,13,14].

In the meantime, it is noteworthy that during state estimation or filtering procedures in distributed sensor network implementations, the sensor network models or the objective plants are likely to experience undesired model inaccuracies or uncertainties, which could lead to certain performance degradation in practical implementations [15,16,17,18,19]. This implies that the designed distributed state estimators or filters that rely on the sensor network could endure certain gain fluctuations, such that the overall stable estimation procedures could be fragile. Therefore, increasing research effort has been made towards the non-fragile design framework [20,21]. Moreover, from the sensor node model point of view, limitations still exist with nonlinear dynamics. As we all know, all practical sensors have nonlinear features, whereas most existing works always assume that sensors only have linear models [22,23,24]. Thus, nonlinearities should be considered for sensor networks with respect to their sensitive models. To the best of our knowledge from recent investigations, certain distributed state estimation with sensor networks subject to sensor nonlinearities and gain fluctuations has not been fully investigated yet and still remains a challenging research topic, which motivates the current study.

To sum up the aforementioned discussions, this paper aims at solving the distributed state estimation problem for uncertain nonlinear systems of sensor networks subject to sensor nonlinearities. Furthermore, non-fragile strategies for enduring the model uncertainties are proposed to improve the sensor model’s robustness. In comparison to most existing studies, our main novelties can be listed as follows:

(1) Firstly, the three essential issues, i.e., the target plant model uncertainties, the state estimator gain variation, and the sensor nonlinearities, are all considered in a unified framework, which approximates the sensor network implementation much more practically. Especially, our work makes one of the first attempts to deal with sensor nonlinearities in the scope of distributed sensor networks.
(2) Secondly, in order to capture the distributed sensor network information exchanges, the model transformation for distributed state estimation errors is performed and new sufficient conditions are established, which leads to the resulting error system being able to achieve a desired passivity performance index from the energy point of view.
(3) Finally, the theoretical derivations and findings are presented in the form of linear matrix inequalities, which can be conveniently calculated with feasible solutions, and the corresponding simulation example is given to verify the effectiveness of our proposed methods.

The remaining parts of our work are listed as follows. Section 2 presents the formulistic description of the distributed state estimation problem. Our theoretical findings along with proof details are derived in Section 3. Section 4 shows an illustrative example by discussions of simulations and Section 5 draws the conclusions and presents our future research prospects.

Notation: $R^{n}$ denotes *n*-dimensional Euclidean space. $R^{m\times n}$ represents a set of m×n real matrices. A matrix M>0 means that *M* is positive definite. ⊗ stands for the Kronecker product. ★ denotes the ellipsis terms in symmetry matrices. $L_{2}\left[0,\infty \right)$ denotes the space of square-integrable vector functions over [0,∞).

## 2. Problem Formulation

### 2.1. Nonlinear Target Plant Model

Consider the following nonlinear system with parameter uncertainties and external disturbances:

(1) $$\left\{\begin{array}{c} \dot{x}\left(t\right)=\left(A+\Delta A\right)x\left(t\right)+f\left(x\left(t\right)\right)+\left(B+\Delta B\right)w\left(t\right),\\ z(t)=(L+\Delta L)x(t),\\ x\left(t\right)=x_{0}, \end{array}\right.$$

where $x\left(t\right)\in R^{n}$ denotes the target plant state, $w\left(t\right)\in R^{n}$ represents the external environmental disturbance that belongs to $L_{2}\left[0,\infty \right)$, and $z\left(t\right)\in R^{l}$ stands for the target plant output measurement to be estimated by the sensor network. Especially, f(∗) stands for the nonlinear function satisfying the sector condition by

(2) $$\begin{array}{c} {\left(f\left(x\left(t\right)\right)-F_{1}x\left(t\right)\right)}^{T}\left(f\left(x\left(t\right)\right)-F_{2}x\left(t\right)\right)\leq 0, \end{array}$$

where $F_{1}$ and $F_{2}$ are the real matrices with $F=F_{2}-F_{1}>0$, respectively [25].

For the system matrices, *A*, B, and *L* are known constant system matrices, and ΔA, ΔB, and ΔL are the system parameter uncertainties, respectively, where

(3) $$\begin{array}{cc} \Delta A= & M_{A}\Phi \left(t\right)N_{A}, \end{array}$$

(4) $$\begin{array}{cc} \Delta B= & M_{B}\Phi \left(t\right)N_{B}, \end{array}$$

(5) $$\begin{array}{cc} \Delta L= & M_{L}\Phi \left(t\right)N_{L}. \end{array}$$

where $M_{A}$, $M_{B}$, $M_{L}$, $N_{A}$, $N_{B}$, and $N_{L}$ are known constant matrices and Φ(t) is an unknown time-varying matrix satisfying

(6) $$\Phi^{T}\left(t\right)\Phi \left(t\right)\leq I.$$

### 2.2. Distributed Sensor Network

The sensor network group of *N* sensor nodes is constructed in the distributed communication fashion. Within this communication framework, the network topology is modeled and denoted by a directed graph; G={V,E,A} and V={1,…,N} where $V\left(G\right)=\left\{v_{1},\ldots ,v_{N}\right\}$ and E are the sets of nodes and edges, and $A=\left[a_{ij}\right]\in R^{N\times N}$ is the weighted adjacency matrix, i.e.,

(7) $$A={\left[a_{ij}\right]}_{N}$$

means that

$$A=\left[\begin{array}{ccccc} a_{11} & a_{12} & \ldots & a_{1(N-1)} & a_{1N}\\ a_{21} & a_{22} & \ldots & a_{2(N-1)} & a_{2N}\\ \vdots & \vdots & \ddots & \vdots & \vdots \\ a_{(N-1)1} & a_{(N-1)2} & \ldots & a_{\left(N-1)(N-1\right)} & a_{(N-1)N}\\ a_{N1} & a_{N2} & \ldots & a_{N(N-1)} & a_{NN} \end{array}\right]$$

and $N_{i}$ presents the local neighbors of sensor *i*. More precisely, assume that $a_{ij}>0$ if sensor *i* can receive certain signals from sensor *j* with $a_{ii}=1$ and $a_{ij}=0$ if there are no signal transmissions between sensor *i* and sensor *j* [26,27].

Furthermore, all the sensors share a unified sampling sequel for satisfying clock synchronization and the time-varying sampling period defined by $t_{k+1}-t_{k}=h\left(t\right)>0,k=0,1,2,\ldots$. In addition, it is assumed that $h\left(t\right)\leq \bar{h}$, where $\bar{h}$ is a known constant.

Consequently, the sampled data measurement for sensor *i* can be represented as follows:

(8) $$\begin{array}{c} y_{i}\left(t\right)=C_{i}x\left(t_{k}\right)+D_{i}w\left(t\right),i=1,2,\ldots ,N, \end{array}$$

where $y_{i}\left(t\right)$ stands for the *i*th sensor measurement output and $C_{i}$ and $D_{i}$ denote the constant system matrix, respectively.

Furthermore, consider the practical measurement of sensors; nonlinear features are often observed on the sensors. As a consequence, the measurement for sensor *i* has the sensor nonlinearities

(9) $$\begin{array}{c} y_{i}\left(t\right)=g\left(C_{i}x\left(t_{k}\right)\right)+D_{i}w\left(t\right),i=1,2,\ldots ,N. \end{array}$$

where the nonlinear function g(∗) also satisfies a sector condition as follows:

(10) $$\begin{array}{c} {\left(g\left(x\left(t\right)\right)-G_{1}x\left(t\right)\right)}^{T}\left(g\left(x\left(t\right)\right)-G_{2}x\left(t\right)\right)\leq 0, \end{array}$$

where $G_{1}$ and $G_{2}$ mean the real matrices, respectively.

**Remark** **1.** *It is noticed that the sensor nonlinearities are more general cases for sensor measurement models in practical implementations, since most of the real-world sensors are with a nonlinear structure or dynamical measurement features. Therefore, critical assumptions about the linear models of sensors could lead to considerable conservativeness when applying the sensor network measurement. Although there are several research results on the nonlinear measurement model of a single sensor when dealing with the state estimation problems of control systems, the adoption of nonlinear measurement models for sensor networks still remains an open area. As a consequence, it is reasonable to discuss the effect of sensor nonlinearity by uncontrollable elements or aggressive conditions for distributed sensor networks, which can lead to more applicability and less conservativeness in state estimator designs.*

**Remark** **2.** *It should be pointed out that when all the sensors in the sensor network are in the distributed topology structure via the communication network, the distributed state estimation problem can be solved by the optimal solutions of desired distributed state estimator designs. As a result, the distributed state estimation with a collective of state estimators is utilized instead of a single state estimator, where information exchanges are conducted to reduce the communication and computation resources. Compared with traditional state estimation, distributed state estimation strategies accordingly have the advantages of scalability and robustness.*

Without a loss of generality, both nonlinear functions f(x(t)) and g(x(t)) could be decomposed into linear and nonlinear parts by the following:

(11) $$f\left(x\left(t\right)\right)=F_{1}x\left(t\right)+f_{s}\left(x\left(t\right)\right),$$

where the nonlinearity $f_{s}\left(x\left(t\right)\right)$ belongs to the set $\Omega_{f}$ given by

(12) $$\Omega_{f}=\left\{f_{s}\left(x\left(t\right)\right):f_{s}{\left(x\left(t\right)\right)}^{T}\left(f_{s}\left(x\left(t\right)\right)-Fx\left(t\right)\right)\leq 0\right\}$$

with $F=F_{2}-F_{1}>0,$ and

(13) $$g\left(x\left(t\right)\right)=G_{1}x\left(t\right)+g_{s}\left(x\left(t\right)\right),$$

where the nonlinearity $g_{s}\left(x\left(t\right)\right)$ belongs to the set $\Omega_{g}$ given by

(14) $$\Omega_{g}=\left\{g_{s}\left(x\left(t\right)\right):g_{s}{\left(x\left(t\right)\right)}^{T}\left(g_{s}\left(x\left(t\right)\right)-Gx\left(t\right)\right)\leq 0\right\}$$

with $G=G_{2}-G_{1}>0.$

**Figure 1:**
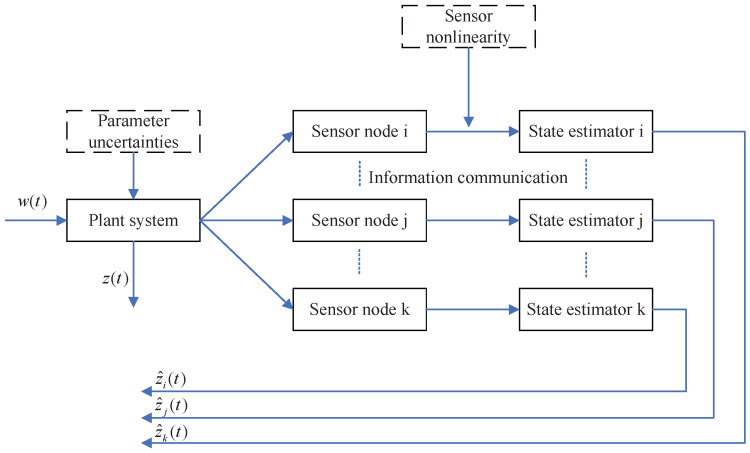
Distributed state estimation in sensor network.

With the above measurement of the sensor network, the corresponding distributed state estimator to the target plant output could be represented from the control theory point of view as follows:

(15) $$\left\{\begin{array}{c} {\dot{\hat{x}}}_{i}\left(t\right)=A{\hat{x}}_{i}\left(t\right)+f\left({\hat{x}}_{i}\left(t\right)\right)\\ \;+\sum_{j\in N_{i}}a_{ij}K_{ij}\left(C_{j}{\hat{x}}_{j}\left(t_{k}\right)-y_{j}\left(t\right)\right),\\ {\hat{z}}_{i}\left(t\right)=L{\hat{x}}_{i}\left(t\right), \end{array}\right.$$

where ${\hat{x}}_{i}\left(t\right)$ represents the estimation of x(t) by sensor *i*, ${\hat{z}}_{i}\left(t\right)$ represents the output measurement of x(t) by sensor *i*, and $K_{ij}$ denotes the distributed state estimator gain to be designed properly. The overall system framework can be seen in Figure 1.

In addition, considering the fact that the gain fluctuation $\Delta K_{ij}$ has certain practical implementations, the above distributed state estimators could be reformulated as follows:

(16) $$\left\{\begin{array}{c} {\dot{\hat{x}}}_{i}\left(t\right)=A{\hat{x}}_{i}\left(t\right)+f\left({\hat{x}}_{i}\left(t\right)\right)\\ \;+\sum_{j\in N_{i}}a_{ij}\left(K_{ij}+\Delta K_{ij}\right)\left(C_{j}{\hat{x}}_{j}\left(t_{k}\right)-g\left(C_{j}x\left(t_{k}\right)\right)-D_{j}w\left(t\right)\right),\\ {\hat{z}}_{i}\left(t\right)=L{\hat{x}}_{i}\left(t\right), \end{array}\right.$$

where $\Delta K_{ij}=M_{ij}\Omega \left(t\right)N$ with $\Omega^{T}\left(t\right)\Omega \left(t\right)\leq I$; $M_{ij}$ and *N* are known constant matrices.

Aiming at the state estimation error of sensor *i*, the following state dynamics can be introduced:

(17) $$\begin{array}{cc} \hat{x}\left(t\right) & ={\left[{\hat{x}}_{1}^{T}\left(t\right),{\hat{x}}_{2}^{T}\left(t\right),\ldots ,{\hat{x}}_{N}^{T}\left(t\right)\right]}^{T}, \end{array}$$

(18) $$\begin{array}{cc} \bar{x}\left(t\right) & =I_{N}⊗x\left(t\right), \end{array}$$

(19) $$\begin{array}{cc} \hat{z}\left(t\right) & ={\left[{\hat{z}}_{1}^{T}\left(t\right),{\hat{z}}_{2}^{T}\left(t\right),\ldots ,{\hat{z}}_{N}^{T}\left(t\right)\right]}^{T}, \end{array}$$

(20) $$\begin{array}{cc} \bar{z}\left(t\right) & =I_{N}⊗z\left(t\right), \end{array}$$

Then, by further letting $\eta \left(t\right)={\left[{\bar{x}}^{T}\left(t\right),{\hat{x}}^{T}\left(t\right)\right]}^{T}$ and $\epsilon \left(t\right)=\bar{z}\left(t\right)-\hat{z}\left(t\right)$, the following augmented state estimation dynamics can be obtained:

(21) $$\left\{\begin{array}{c} \dot{\eta }\left(t\right)=\left(A+F_{1}\right)\eta \left(t\right)+\left(\mathrm{KC}-{\mathrm{KG}}_{1}\right)\eta \left(t_{k}\right)+F_{s}\left(\eta \left(t\right)\right)-{\mathrm{KG}}_{s}\left(\eta \left(t_{k}\right)\right)+\left(B-\mathrm{KD}\right)w\left(t\right),\\ \epsilon (t)=L\eta (t), \end{array}\right.$$

where

$$\begin{array}{cc} A= & \left[\begin{array}{cc} \left(I_{N}⊗A+I_{N}⊗\Delta A\right) & 0\\ 0 & \left(I_{N}⊗A\right) \end{array}\right],\\ F(\eta (t))= & \left[\begin{array}{c} \bar{f}\left(x\left(t\right)\right)\\ \hat{f}\left({\hat{x}}_{1}\left(t\right)\right) \end{array}\right],\\ F_{1} & =I_{2N}⊗F_{1},\\ B= & \left[\begin{array}{c} \left(\bar{B}+\Delta \bar{B}\right)\\ 0 \end{array}\right],\\ K= & \left[\begin{array}{cc} 0 & 0\\ 0 & \hat{K}+\Delta \hat{K} \end{array}\right],\\ C= & \left[\begin{array}{cc} 0 & 0\\ 0 & \hat{C} \end{array}\right],\\ D= & \left[\begin{array}{c} 0\\ \hat{D} \end{array}\right],\\ L= & \left[\begin{array}{cc} \left(\bar{L}+\Delta \bar{L}\right) & -\bar{L}) \end{array}\right],\\ G(\eta (t))= & \left[\begin{array}{c} 0\\ \hat{g}\left(x\left(t\right)\right) \end{array}\right],\\ G_{1}= & I_{2N}⊗G_{1}, \end{array}$$

with

$$\begin{array}{cc} \bar{B}= & {\left[B^{T},B^{T},\ldots ,B^{T}\right]}_{N}^{T},\\ \hat{K}= & {\left[a_{ij}K_{ij}\right]}_{N\times N},\\ \hat{C}= & \mathrm{diag}\{C_{1},C_{2},\ldots ,C_{N}\},\\ \hat{D}= & {\left[C_{1}^{T},C_{2}^{T},\ldots ,C_{N}^{T}\right]}^{T},\\ \bar{L}= & {\left[L,L,\ldots ,L\right]}_{N},\\ \bar{f}\left(x\left(t\right)\right)= & {\left[f^{T}\left(x\left(t\right)\right),f^{T}\left(x\left(t\right)\right),\ldots ,f^{T}\left(x\left(t\right)\right)\right]}_{N}^{T},\\ \hat{f}\left({\hat{x}}_{1}\left(t\right)\right)= & {\left[f^{T}\left(x_{1}\left(t\right)\right),f^{T}\left(x_{2}\left(t\right)\right),\ldots ,f^{T}\left(x_{N}\left(t\right)\right)\right]}_{N}^{T},,\\ \hat{g}\left(x\left(t\right)\right)= & {\left[g^{T}\left(C_{1}x\left(t\right)\right),g^{T}\left(C_{2}x\left(t\right)\right),\ldots ,g^{T}\left(C_{N}x\left(t\right)\right)\right]}_{N}^{T},\\ F(\eta (t))= & F_{1}\eta \left(t\right)+F_{s}\left(\eta \left(t\right)\right),\\ G(\eta (t))= & G_{1}\eta \left(t\right)+G_{s}\left(\eta \left(t\right)\right). \end{array}$$

The purpose of this paper is to design the desired distributed state estimator gains to ensure the following passivity performance in the sensor network, such that the target plant output measurement can be well estimated despite the effect of disturbances.

**Definition** **1** ([28])**.** *Under the zero initial condition, the passivity-based performance index γ for the above augmented state estimation dynamics is said to be achieved if for all $t_{p}>0$ and any non-zero value it holds that*

(22) $$\int_{0}^{t_{p}}2\epsilon^{T}\left(s\right)w\left(s\right)ds\geq -\gamma \int_{0}^{t_{p}}w^{T}\left(s\right)w\left(s\right)ds.$$

**Remark** **3.** *The passivity-based performance is another important disturbance attenuation performance index that is different from the $H_{\infty }$ performance. It is focused on the energy point of view and is related to the system input and system output, which means that the energy increment should be less than that supplied for the system while accordingly guaranteeing system stability.*

To this end, the following important lemma is introduced for deriving the main results.

**Lemma** **1** ([29])**.** *Given the matrices $X^{T}=X$ and Y, Z with appropriate dimensions and ϝ*(*t*)* satisfying ϝ^T^*(*t*)*ϝ*(*t*)* ≤ I, it holds that X+Yϝ(t)Z+(Yϝ*(*t*)*$Z{)}^{T}<0$, if and only if there exists a scalar e>0 such that*

$$\left[\begin{array}{ccc} X & Y & \varepsilon Z^{T}\\ ★ & -eI & 0\\ ★ & ★ & -eI \end{array}\right]<0.$$

## 3. Main Results

In the following section, effective convex optimization conditions are established on the above distributed state estimator gains with detailed proofs.

**Theorem** **1.** *With the parameter $\bar{h}$, the resulting augmented state estimation dynamics can achieve the passivity performance γ with the given distributed state estimator gains $K_{ij}$, i,j=1,2,…,N, if there exist the matrices P>0, Q>0, and R>0, such that the following convex optimization condition Θ<0 can hold, where*

$$\begin{array}{cc} \Theta = & \left[\begin{array}{cc} \Theta_{1} & \Theta_{2}\\ ★ & \Theta_{3} \end{array}\right],\\ \Theta_{1}= & \left[\begin{array}{ccc} 2P(A+F_{1})+Q-R & P(\mathrm{KC}-{\mathrm{KG}}_{1})+R & 0\\ ★ & -2R & R\\ ★ & ★ & -Q-R \end{array}\right],\\ \Theta_{2}= & \left[\begin{array}{cccc} P+F & -\mathrm{PK}+G & P\left(B-\mathrm{KD}\right)-L^{T}\; & \bar{h}{\left(A+F_{1}\right)}^{T}R\\ 0 & 0 & 0 & \bar{h}{\left(\mathrm{KC}-{\mathrm{KG}}_{1}\right)}^{T}R\\ 0 & 0 & 0 & 0 \end{array}\right],\\ \Theta_{3}= & \left[\begin{array}{cccc} -2I & 0 & 0 & \bar{h}R\\ ★ & -2I & 0 & -\bar{h}K^{T}R\\ ★ & ★ & -\gamma I & \bar{h}{\left(B-\mathrm{KD}\right)}^{T}R\\ ★ & ★ & ★ & -R \end{array}\right]. \end{array}$$

**Proof.**  Firstly, the so-called virtual delay method is adopted, such that one has that

(23) $$\left\{\begin{array}{c} \dot{\eta }\left(t\right)=\left(A+F_{1}\right)\eta \left(t\right)+\left(\mathrm{KC}-{\mathrm{KG}}_{1}\right)\eta \left(t-d\left(t\right)\right)+F_{s}\left(\eta \left(t\right)\right)-{\mathrm{KG}}_{s}\left(\eta \left(t-d\left(t\right)\right)\right)+\left(B-\mathrm{KD}\right)w\left(t\right),\\ \epsilon (t)=L\eta (t), \end{array}\right.$$

where $0\leq d\left(t\right)=t-t_{k}\leq \bar{h}$.

Secondly, we construct the proper Lyapunov–Krasovskii functional as follows:

(24) $$V\left(t\right)=V_{1}\left(t\right)+V_{2}\left(t\right)+V_{3}\left(t\right),$$

where

$$\begin{array}{cc} \; & V_{1}\left(t\right)=\eta^{T}\left(t\right)P\eta \left(t\right),\\ \; & V_{2}\left(t\right)=\int_{t-\bar{h}}^{t}\eta^{T}\left(\varphi \right)Q\eta \left(\varphi \right)d\varphi ,\\ \; & V_{3}\left(t\right)=\bar{h}\int_{-\bar{h}}^{0}\int_{t+\varphi }^{t}{\dot{\eta }}^{T}\left(\eta \right)R\dot{\eta }\left(\eta \right)d\eta d\varphi . \end{array}$$

Consequently, it can be derived by the evolution of V(t) that

(25) $$\begin{array}{cc} {\dot{V}}_{1}\left(t\right)= & 2\eta^{T}\left(t\right)P\dot{\eta }\left(t\right)\\ = & 2\eta^{T}\left(t\right)P\left(A+F_{1}\right)\eta \left(t\right)+\left(\mathrm{KC}-{\mathrm{KG}}_{1}\right)\eta \left(t-d\left(t\right)\right)+F_{s}\left(\eta \left(t\right)\right) \end{array}$$

(26) $$\begin{array}{cc} \; & -{\mathrm{KG}}_{s}\left(\eta \left(t-d\left(t\right)\right)\right)+\left(B-\mathrm{KD}\right)w\left(t\right), \end{array}$$

(27) $$\begin{array}{cc} {\dot{V}}_{2}\left(t\right)= & \eta^{T}\left(t\right)Q\eta \left(t\right)-\eta^{T}\left(t-\bar{h}\right)Q\eta \left(t-\bar{h}\right), \end{array}$$

(28) $$\begin{array}{cc} {\dot{V}}_{3}\left(t\right)= & {\bar{h}}^{2}{\dot{\eta }}^{T}\left(t\right)R\dot{\eta }\left(t\right)-\bar{h}\int_{t-\bar{h}}^{t}{\dot{\eta }}^{T}\left(\varphi \right)R\dot{\eta }\left(\varphi \right)d\varphi . \end{array}$$

Moreover, it can be verified by the nonlinear functions f(x(t)) and g(x(t)) from the augmented state estimation dynamical system that

(29) $$\begin{array}{ccc} F_{s}^{T}\left(\eta \left(t\right)\right)\left(F_{s}\left(\eta \left(t\right)\right)-F\eta \left(t\right)\right) & \leq & 0, \end{array}$$

(30) $$\begin{array}{ccc} G_{s}^{T}\left(\eta \left(t\right)\right)\left(G_{s}\left(\eta \left(t\right)\right)-G\eta \left(t\right)\right) & \leq & 0, \end{array}$$

with $F=(I_{N}⊗F)$ and G= $\left(I_{N}⊗G\right)$, such that it holds that

(31) $$G_{s}^{T}\left(\eta \left(t-d\left(t\right)\right)\right)\left(G_{s}\left(\eta \left(t-d\left(t\right)\right)\right)-G\eta \left(t-d\left(t\right)\right)\right)\leq 0.$$

On the other hand, one has that

(32) $$\begin{array}{cc} \; & {\bar{h}}^{2}{\dot{\eta }}^{T}\left(t\right)R\dot{\eta }\left(t\right)\\ \; & =\zeta^{T}\left(t\right)\left[\begin{array}{c} \bar{h}{\left(A+F_{1}\right)}^{T}\\ \bar{h}{\left(\mathrm{KC}-{\mathrm{KG}}_{1}\right)}^{T}\\ 0\\ \bar{h}I\\ -\bar{h}K^{T}\\ \bar{h}{\left(B-\mathrm{KD}\right)}^{T} \end{array}\right]R{\left[\begin{array}{c} \bar{h}{\left(A+F_{1}\right)}^{T}\\ \bar{h}{\left(\mathrm{KC}-{\mathrm{KG}}_{1}\right)}^{T}\\ 0\\ \bar{h}I\\ -\bar{h}K^{T}\\ \bar{h}{\left(B-\mathrm{KD}\right)}^{T} \end{array}\right]}^{T}\zeta \left(t\right), \end{array}$$

where $\zeta \left(t\right)={\left[\eta^{T}\left(t\right),\eta^{T}\left(t-d\left(t\right)\right),\eta^{T}\left(t-\bar{h}\right),F_{s}^{T}\left(\eta \left(t\right)\right),G_{s}^{T}\left(\eta \left(t-d\left(t\right)\right)\right),w^{T}\left(t\right)\right]}^{T}.$

Furthermore, it can be deduced by Jensen’s inequality lemma [30] that

(33) $$\begin{array}{cc} \; & -\bar{h}\int_{t-\bar{h}}^{t}{\dot{\eta }}^{T}\left(\varphi \right)R\dot{\eta }\left(\varphi \right)d\varphi \\ \leq & {\left[\begin{array}{c} \eta (t)\\ \eta (t-d(t))\\ \eta (t-\bar{h}) \end{array}\right]}^{T}\left[\begin{array}{ccc} -R & R & 0\\ ★ & -2R & R\\ ★ & ★ & -R \end{array}\right]\left[\begin{array}{c} \eta (t)\\ \eta (t-d(t))\\ \eta (t-\bar{h}) \end{array}\right]. \end{array}$$

For the passivity performance index, it can be defined that

(34) $$J_{p}=\int_{0}^{t_{p}}\left[-2\epsilon^{T}\left(t\right)w\left(t\right)-\gamma w^{T}\left(t\right)w\left(t\right)\right]dt.$$

such that it can be derived that

(35) $$\begin{array}{cc} \; & \dot{V}\left(t\right)-2F_{s}^{T}\left(\eta \left(t\right)\right)\left(F_{s}\left(\eta \left(t\right)\right)+\left(I_{N}⊗F\right)\eta \left(t\right)\right)\\ \; & -2G_{s}^{T}\left(\eta \left(t-d\left(t\right)\right)\right)\left(G_{s}\left(\eta \left(t-d\left(t\right)\right)\right)+\left(I_{N}⊗G\right)\eta \left(t-d\left(t\right)\right)\right)\\ \; & -2\epsilon^{T}\left(t\right)w\left(t\right)-\gamma w^{T}\left(t\right)w\left(t\right)\\ \leq & \zeta^{T}\left(t\right)\left(\bar{\Theta }+\left[\begin{array}{c} \bar{h}{\left(A+F_{1}\right)}^{T}\\ \bar{h}{\left(\mathrm{KC}-{\mathrm{KG}}_{1}\right)}^{T}\\ 0\\ \bar{h}I\\ -\bar{h}K^{T}\\ \bar{h}{\left(B-\mathrm{KD}\right)}^{T} \end{array}\right]R{\left[\begin{array}{c} \bar{h}{\left(A+F_{1}\right)}^{T}\\ \bar{h}{\left(\mathrm{KC}-{\mathrm{KG}}_{1}\right)}^{T}\\ 0\\ \bar{h}I\\ -\bar{h}K^{T}\\ \bar{h}{\left(B-\mathrm{KD}\right)}^{T} \end{array}\right]}^{T}\right)\zeta \left(t\right), \end{array}$$

where

$$\begin{array}{cc} \bar{\Theta }= & \left[\begin{array}{cc} {\bar{\Theta }}_{1} & {\bar{\Theta }}_{2}\\ ★ & {\bar{\Theta }}_{3} \end{array}\right],\\ {\bar{\Theta }}_{1}= & \left[\begin{array}{cc} 2P(A+F_{1})+Q-R & P(\mathrm{KC}-{\mathrm{KG}}_{1})+R\\ ★ & -2R \end{array}\right],\\ {\bar{\Theta }}_{2}= & \left[\begin{array}{cccc} 0 & P+F & -\mathrm{PK}+G & P\left(B-\mathrm{KD}\right)-L^{T}\\ R & 0 & 0 & 0 \end{array}\right],\\ {\bar{\Theta }}_{3}= & \left[\begin{array}{cccc} -Q-R & 0 & 0 & 0\\ ★ & -2I & 0 & 0\\ ★ & ★ & -2I & 0\\ ★ & ★ & ★ & -\gamma I \end{array}\right]. \end{array}$$

Finally, by applying the Schur complement lemma, it can be verified that when Θ<0 holds under zero initial conditions, it can ensure that $J_{p}<0$, which means that the passivity performance γ could be satisfied according to Definition 1 and therefore completes the proof. □

**Remark** **4.** *The virtual delay method is a very important model transformation strategy when dealing with the sampled data control system or networked control systems. More precisely, for the sampling state $x(t_{k})$, it can be obtained that*

$$x\left(t_{k}\right)=x\left(t-h\left(t\right)\right),t_{k}\leq t<t_{k+1},$$

*where a time-varying input delay, $h\left(t\right)=t-t_{k}$, is called the virtual delay. Then, the dynamical system model can be converted to the continuous-time model for control system analysis and synthesis.*

The above established convex optimization conditions are not standard LMIs, such that the following theorem is further presented for the state estimator gains design in the form of strict LMIs.

**Theorem** **2.** *With the parameter $\bar{h}$, the resulting augmented state estimation dynamics can achieve the passivity performance γ, if there exist the matrices $\mathrm{diag}\{P_{1},P_{2}\}>0$ with $P_{1}=I_{N}⊗P_{1}\in R^{N\times n},$ $P_{2}=\mathrm{diag}\left\{P_{2},P_{3},\ldots ,P_{N+1}\right\}$ $\in R^{N\times n}$, $Q=\mathrm{diag}\{Q_{1},Q_{2}\}>0$ with $Q_{1}=I_{N}⊗Q_{1}\in R^{N\times n},$ $Q_{2}=I_{N}⊗Q_{2}\in R^{N\times n}$, $R=\mathrm{diag}\{R_{1},R_{2}\}>0$ with $R_{1}=I_{N}⊗R_{1}\in R^{N\times n},$ $R_{2}=I_{N}⊗R_{2}\in R^{N\times n}$, and $W\in R^{N\times n}$, such that the following convex optimization condition $\bar{\Theta }<0$ can hold, where*

$$\begin{array}{cc} \bar{\Theta }= & \left[\begin{array}{cc} {\bar{\Theta }}_{1} & {\bar{\Theta }}_{2}\\ ★ & {\bar{\Theta }}_{3} \end{array}\right],\\ {\bar{\Theta }}_{1}= & \left[\begin{array}{cc} {\bar{\Theta }}_{11} & {\bar{\Theta }}_{12}\\ ★ & {\bar{\Theta }}_{13} \end{array}\right],\\ {\bar{\Theta }}_{11}= & \left[\begin{array}{cccc} {\bar{\Theta }}_{111} & 0 & R_{1} & 0\\ ★ & {\bar{\Theta }}_{112} & 0 & W\left(\hat{C}-I_{N}⊗G_{1}\right)+R_{2}\\ ★ & ★ & -2R_{1} & 0\\ ★ & ★ & ★ & -2R_{2} \end{array}\right],\\ {\bar{\Theta }}_{111}= & 2P_{1}\left(I_{N}⊗A\right)+I_{N}⊗F_{1}+Q_{1}-R_{1},\\ {\bar{\Theta }}_{112}= & 2P_{2}\left(I_{N}⊗A\right)+I_{N}⊗F_{1}+Q_{2}-R_{2},\\ {\bar{\Theta }}_{12}= & \left[\begin{array}{cccccc} 0 & 0 & P_{1}+I_{N}⊗F_{1} & 0 & I_{N}⊗G_{1} & 0\\ 0 & 0 & 0 & P_{2}+I_{N}⊗F_{1} & 0 & -W+I_{N}⊗G_{1}\\ R_{1} & 0 & 0 & 0 & 0 & 0\\ 0 & R_{2} & 0 & 0 & 0 & 0 \end{array}\right], \end{array}$$

$$\begin{array}{cc} {\bar{\Theta }}_{13}= & \left[\begin{array}{cccccc} -Q_{1}-R_{1} & 0 & 0 & 0 & 0 & 0\\ ★ & -Q_{2}-R_{2} & 0 & 0 & 0 & 0\\ ★ & ★ & -2I & 0 & 0 & 0\\ ★ & ★ & ★ & -2I & 0 & 0\\ ★ & ★ & ★ & ★ & -2I & 0\\ ★ & ★ & ★ & ★ & ★ & -2I \end{array}\right],\\ {\bar{\Theta }}_{2}= & \left[\begin{array}{ccc} {\bar{\Theta }}_{21} & {\bar{\Theta }}_{22} & {\bar{\Theta }}_{23} \end{array}\right],\\ {\bar{\Theta }}_{21}= & \left[\begin{array}{ccc} P_{1}\bar{B}-{\left(I_{N}⊗L\right)}^{T} & \bar{h}{\left(I_{N}⊗A\right)}^{T}P_{1}+\bar{h}{\left(I_{N}⊗F_{1}\right)}^{T}P_{1} & 0\\ -W\hat{D}+{\left(I_{N}⊗L\right)}^{T} & 0 & \bar{h}{\left(I_{N}⊗A\right)}^{T}P_{2}+{\left(I_{N}⊗F_{1}\right)}^{T}P_{2}\\ 0 & 0 & 0\\ 0 & 0 & \bar{h}{\hat{C}}^{T}W^{T}-h{\left(I_{N}⊗G_{1}\right)}^{T}W^{T}\\ 0 & 0 & 0\\ 0 & 0 & 0\\ 0 & \bar{h}P_{1} & 0\\ 0 & 0 & \bar{h}P_{2}\\ 0 & 0 & 0\\ 0 & 0 & -\bar{h}W^{T} \end{array}\right],\\ {\bar{\Theta }}_{22}= & \left[\begin{array}{cccc} P_{1}\left(I_{N}⊗M_{A}\right) & P_{1}\left(I_{N}⊗M_{B}\right) & 0\; & 0\\ 0 & 0 & P_{2}M_{K} & 0\\ 0 & 0 & 0 & 0\\ 0 & 0 & 0 & 0\\ 0 & 0 & 0 & 0\\ 0 & 0 & 0 & 0\\ 0 & 0 & 0 & 0\\ 0 & 0 & 0 & 0\\ 0 & 0 & 0 & 0\\ 0 & 0 & 0 & 0 \end{array}\right], \end{array}$$

$$\begin{array}{cc} {\bar{\Theta }}_{23}= & \left[\begin{array}{cccc} {\left(I_{N}⊗N_{A}\right)}^{T} & 0 & 0 & N_{L}^{T}\\ 0 & 0 & 0 & 0\\ 0 & 0 & 0 & 0\\ 0 & 0 & {\left(\hat{C}-I_{N}⊗G_{1}\right)}^{T}N_{K}^{T} & 0\\ 0 & 0 & 0 & 0\\ 0 & 0 & 0 & 0\\ 0 & 0 & 0 & 0\\ 0 & 0 & 0 & 0\\ 0 & 0 & 0 & 0\\ 0 & 0 & -N_{K}^{T} & 0 \end{array}\right],\\ {\bar{\Theta }}_{3}= & \left[\begin{array}{cc} {\bar{\Theta }}_{31} & {\bar{\Theta }}_{32}\\ ★ & {\bar{\Theta }}_{33} \end{array}\right],\\ {\bar{\Theta }}_{31}= & \left[\begin{array}{ccc} -\gamma I & \bar{h}{\bar{B}}^{T}P_{1} & -\bar{h}{\hat{D}}^{T}{\hat{K}}^{T}P_{2}\\ ★ & R_{1}-2P_{1} & 0\\ ★ & ★ & R_{2}-2P_{2} \end{array}\right],\\ {\bar{\Theta }}_{32}= & \left[\begin{array}{cccccccc} 0 & 0 & 0 & -M_{L} & 0 & 0 & 0 & 0\\ \bar{h}P_{1}\left(I_{N}⊗M_{A}\right) & \bar{h}P_{1}\left(I_{N}⊗M_{B}\right) & 0 & 0 & 0 & 0 & 0 & 0\\ 0 & 0 & \bar{h}P_{2}M_{K} & 0 & 0 & 0 & 0 & 0 \end{array}\right],\\ {\bar{\Theta }}_{33}= & \mathrm{diag}\{-eI,-eI,-eI,-eI,-eI,-eI,-eI,-eI\}. \end{array}$$

*Furthermore, the desired distributed state estimator gains $K_{ij}$, i,j=1,2,…N, are designed by W with*

$$\hat{K}=P_{2}^{-1}W.$$

**Proof.**  Firstly, by denoting $W=P_{2}\hat{K}$, it can be derived that

(36) $$\Theta =\tilde{\Theta }+\Theta_{1}\Xi \left(t\right)\Theta_{2}+\Theta_{2}^{T}\Xi \left(t\right)\Theta_{1}^{T},$$

where

$$\begin{array}{cc} \tilde{\Theta }= & \left[\begin{array}{cc} {\tilde{\Theta }}_{1} & {\tilde{\Theta }}_{2}\\ ★ & {\tilde{\Theta }}_{3} \end{array}\right],\\ {\tilde{\Theta }}_{1}= & \left[\begin{array}{cc} {\tilde{\Theta }}_{11} & {\tilde{\Theta }}_{12}\\ ★ & {\tilde{\Theta }}_{13} \end{array}\right],\\ {\tilde{\Theta }}_{11}= & \left[\begin{array}{cccccc} {\tilde{\Theta }}_{111} & 0 & R_{1} & 0 & 0 & 0\\ ★ & {\tilde{\Theta }}_{112} & 0 & 0 & 0 & 0\\ ★ & ★ & -2R_{1} & 0 & R_{1} & 0\\ ★ & ★ & ★ & -2R_{2} & 0 & R_{2}\\ ★ & ★ & ★ & ★ & -Q_{1}-R_{1} & 0 \end{array}\right],\\ {\tilde{\Theta }}_{12}= & \left[\begin{array}{ccc} P_{1}+I_{N}⊗F_{1} & 0 & I_{N}⊗G_{1}\\ 0 & P_{2}+I_{N}⊗F_{1} & 0\\ 0 & 0 & 0\\ 0 & 0 & 0\\ 0 & 0 & 0 \end{array}\right], \end{array}$$

$$\begin{array}{cc} {\tilde{\Theta }}_{13}= & \left[\begin{array}{cccc} -Q_{2}-R_{2} & 0 & 0 & 0\\ ★ & -2I & 0 & 0\\ ★ & ★ & -2I & 0\\ ★ & ★ & ★ & -2I \end{array}\right],\\ {\tilde{\Theta }}_{111}= & 2P_{1}\left(I_{N}⊗A\right)+I_{N}⊗F_{1}+Q_{1}-R_{1},\\ {\tilde{\Theta }}_{112}= & 2P_{2}\left(I_{N}⊗A\right)+I_{N}⊗F_{1}+Q_{2}-R_{2},\\ {\tilde{\Theta }}_{2}= & \left[\begin{array}{cc} {\tilde{\Theta }}_{21} & {\tilde{\Theta }}_{22} \end{array}\right],\\ {\tilde{\Theta }}_{21}= & \left[\begin{array}{cc} 0 & P_{1}\bar{B}-{\left(I_{N}⊗L\right)}^{T}\\ -P_{2}\hat{K}+I_{N}⊗G_{1} & -P_{2}\hat{K}\hat{D}+{\left(I_{N}⊗L\right)}^{T}\\ 0 & 0\\ 0 & 0\\ 0 & 0\\ 0 & 0\\ 0 & 0\\ 0 & 0\\ 0 & 0 \end{array}\right],\\ {\tilde{\Theta }}_{22}= & \left[\begin{array}{cc} \bar{h}{\left(I_{N}⊗A\right)}^{T}P_{1}+\bar{h}{\left(I_{N}⊗F_{1}\right)}^{T}P_{1} & 0\\ 0 & \bar{h}{\left(I_{N}⊗A\right)}^{T}P_{2}+{\left(I_{N}⊗F_{1}\right)}^{T}P_{2}\\ 0 & 0\\ 0 & \bar{h}{\hat{C}}^{T}{\hat{K}}^{T}P_{2}-h{\left(I_{N}⊗G_{1}\right)}^{T}{\hat{K}}^{T}P_{2}\\ 0 & 0\\ 0 & 0\\ \bar{h}P_{1} & 0\\ 0 & \bar{h}P_{2}\\ 0 & 0 \end{array}\right],\\ {\tilde{\Theta }}_{3}= & \left[\begin{array}{cccc} -2I & 0 & 0 & -\bar{h}{\hat{K}}^{T}P_{2}\\ ★ & -\gamma I & \bar{h}{\bar{B}}^{T}P_{1} & -\bar{h}{\hat{D}}^{T}{\hat{K}}^{T}P_{2}\\ ★ & ★ & R_{1}-2P_{1} & 0\\ ★ & ★ & ★ & R_{2}-2P_{2} \end{array}\right],\\ \Theta_{1}= & \left[\begin{array}{cccc} P_{1}\left(I_{N}⊗M_{A}\right) & P_{1}\left(I_{N}⊗M_{B}\right) & 0\; & 0\\ 0 & 0 & P_{2}M_{K} & 0\\ 0 & 0 & 0 & 0\\ 0 & 0 & 0 & 0\\ 0 & 0 & 0 & 0\\ 0 & 0 & 0 & 0\\ 0 & 0 & 0 & 0\\ 0 & 0 & 0 & 0\\ 0 & 0 & 0 & 0\\ 0 & 0 & 0 & 0\\ 0 & 0 & 0 & -M_{L}\\ \bar{h}P_{1}\left(I_{N}⊗M_{A}\right) & \bar{h}P_{1}\left(I_{N}⊗M_{B}\right) & 0 & 0\\ 0 & 0 & \bar{h}P_{2}M_{K} & 0 \end{array}\right], \end{array}$$

$$\begin{array}{cc} \Theta_{2}= & \left[\begin{array}{ccccccccccccc} \left(I_{N}⊗N_{A}\right) & 0 & 0 & 0 & 0 & 0 & 0 & 0 & 0 & 0 & 0 & 0 & 0\\ 0 & 0 & 0 & 0 & 0 & 0 & 0 & 0 & 0 & 0 & \left(I_{N}⊗N_{B}\right) & 0 & 0\\ 0 & 0 & 0 & \Omega N_{K}\left(\hat{C}-I_{N}⊗G_{1}\right) & 0 & 0 & 0 & 0 & 0 & -N_{K} & -\Omega N_{K}\hat{D} & 0 & 0\\ N_{L} & 0 & 0 & 0 & 0 & 0 & 0 & 0 & 0 & 0 & 0 & 0 & 0 \end{array}\right],\\ \Xi (t)= & \mathrm{diag}\{\left(I_{N}⊗\Phi \left(t\right),I_{N}⊗\Phi \left(t\right),\Omega \left(t\right),\Phi \left(t\right),\Phi \left(t\right),\ldots ,\Phi \left(t\right)\right\}. \end{array}$$

Then, by applying Lemma 1 to the above results and recalling that $\Xi^{T}\left(t\right)\Xi \left(t\right)\leq I$, the remainder of the proof can be directly obtained according to Theorem 1. □

**Remark** **5.** *It is worth mentioning that by applying some notable Lyapunov–Krasovskii functionals, less conservative stability results can be derived accordingly. Since the established LMI results are related to the dimension of the system and the number of sensor nodes, the computational complexity tradeoff of convex optimization between the sensor nodes and the feasible design solutions should be then considered.*

## 4. Illustrative Examples

For the following section, the effectiveness of our theoretical results is demonstrated based on two simulation examples.

**Example** **1.** *During the simulation, the following nonlinear dynamical system was presented, where its system parameters were chosen by*

$$\begin{array}{cc} \; & A=\left[\begin{array}{cc} -3.8 & 0.4\\ 0.3 & -4.5 \end{array}\right],\\ \; & B=\left[\begin{array}{c} 0.5\\ 0.4 \end{array}\right],\\ \; & L=\left[\begin{array}{cc} 1 & 0.5 \end{array}\right]. \end{array}$$

*Moreover, the system parameter uncertainty matrices were assumed to be*

$$\begin{array}{cc} \; & M_{A}=\left[\begin{array}{c} 0.1\\ 0.1 \end{array}\right],N_{A}=\left[\begin{array}{cc} 0.5 & 0.5 \end{array}\right],\\ \; & M_{B}=\left[\begin{array}{c} 0.2\\ 0.2 \end{array}\right],N_{B}=0.4,\\ \; & M_{L}=\left[\begin{array}{cc} 0.1 & 0.1 \end{array}\right],N_{L}=0.1.\\ \; & \Phi =\sin t, \end{array}$$

*and the external disturbance was set by w(t)=0.1sin(t).*

*The system nonlinearity was supposed to be*

$$f\left(x\left(t\right)\right)=\frac{F_{2}+F_{1}}{2}x\left(t\right)+\frac{F_{2}-F_{1}}{2}\sin x\left(t\right),$$

*where*

$$\begin{array}{cc} F_{1}= & \mathrm{diag}\{0.25,0.25\},\\ F_{2}= & \mathrm{diag}\{0.8,0.8\}. \end{array}$$

*A group of four sensors was constructed to work collectively within a sensor network, where the sensor parameters were set by*

$$\begin{array}{cc} \; & C_{1}=\left[\begin{array}{cc} 1.15 & 0\\ 0 & 1.15 \end{array}\right],\;C_{2}=\left[\begin{array}{cc} 1.1 & 0\\ 0 & 0.95 \end{array}\right],\\ \; & C_{3}=\left[\begin{array}{cc} 0.95 & 0\\ 0 & 0.95 \end{array}\right],\;C_{4}=\left[\begin{array}{cc} 0.9 & 0\\ 0 & 1 \end{array}\right], \end{array}$$

*and*

$$\begin{array}{cc} D_{1} & =\left[\begin{array}{c} 0.1\\ 0.2 \end{array}\right],\;D_{2}=\left[\begin{array}{c} 0.2\\ 0.2 \end{array}\right],\\ D_{3} & =\left[\begin{array}{c} 0.3\\ 0.4 \end{array}\right],\;D_{4}=\left[\begin{array}{c} 0.3\\ 0.2 \end{array}\right], \end{array}$$

*and the sensor network topology was formulated with*

$$A=\left[\begin{array}{cccc} 1 & 1 & 0 & 0\\ 0 & 1 & 1 & 0\\ 1 & 0 & 1 & 0\\ 0 & 1 & 0 & 1 \end{array}\right]$$

*and the time-varying sampling periods of the sensors were assumed to be h=0.2 s and h=0.25 s.*

*In addition, the sensor nonlinearities were supposed to be*

$$g\left(x\left(t\right)\right)=\frac{G_{2}+G_{1}}{2}x\left(t\right)+\frac{G_{2}-G_{1}}{2}\cos10x\left(t\right),$$

*where*

$$\begin{array}{cc} G_{1}= & \mathrm{diag}\{0.3,0.3\},\\ G_{2}= & \mathrm{diag}\{0.7,0.8\}. \end{array}$$

*For the distributed state estimator gain fluctuations, it was assumed that*

$$\begin{array}{cc} \; & M_{ij}=\left[\begin{array}{cc} 0.1 & 0.1\\ 0.1 & 0.1 \end{array}\right],i,j=1,2,3,4. \end{array}$$

*and*

$$\begin{array}{c} \Omega =\cos t,\\ N=0.2. \end{array}$$

*For the passivity performance, it was assumed that γ=2. As a result, the desired state estimator gains $K_{ij}$ were calculated by solving the LMIs in Theorem 2 with the following feasible solutions:*

$$\begin{array}{cc} K_{11} & =\left[\begin{array}{cc} -0.1925 & 0.0741\\ 0.0449 & -0.2369 \end{array}\right],\\ K_{12} & =\left[\begin{array}{cc} 0.1070 & 0.1076\\ 0.0623 & 0.0625 \end{array}\right],\\ K_{22} & =\left[\begin{array}{cc} -0.1451 & 0.1057\\ 0.0651 & -0.2012 \end{array}\right], \end{array}$$

$$\begin{array}{cc} K_{23} & =\left[\begin{array}{cc} 0.1372 & 0.1375\\ 0.0824 & 0.0823 \end{array}\right],\\ K_{31} & =\left[\begin{array}{cc} 0.0659 & 0.0668\\ 0.0405 & 0.0409 \end{array}\right],\\ K_{33} & =\left[\begin{array}{cc} -0.0779 & 0.1546\\ 0.0972 & -0.1508 \end{array}\right],\\ K_{42} & =\left[\begin{array}{cc} 0.0959 & 0.0962\\ 0.0594 & 0.0594 \end{array}\right],\\ K_{44} & =\left[\begin{array}{cc} -0.0337 & 0.1780\\ 0.1140 & -0.1126 \end{array}\right]. \end{array}$$

**Figure 2:**
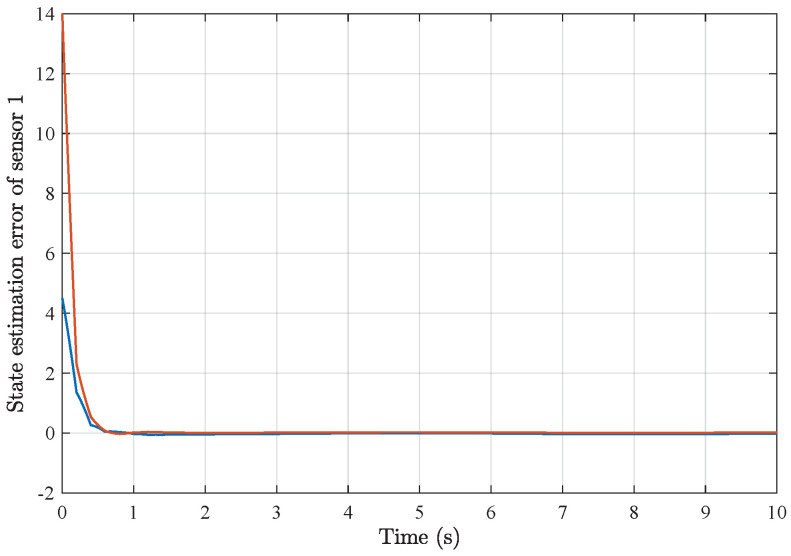
State estimation error of sensor node 1 with 4 sensors.

**Figure 3:**
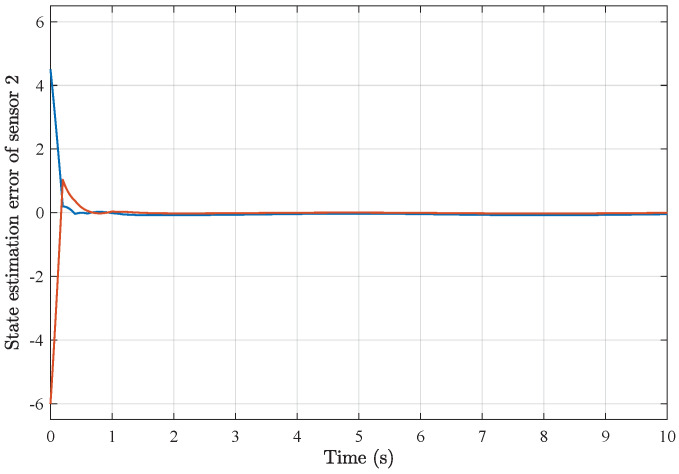
State estimation error of sensor node 2 with 4 sensors.

**Figure 4:**
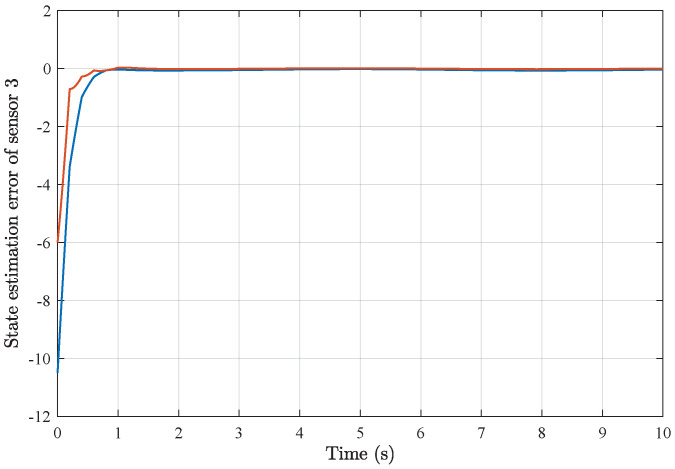
State estimation error of sensor node 3 with 4 sensors.

**Figure 5:**
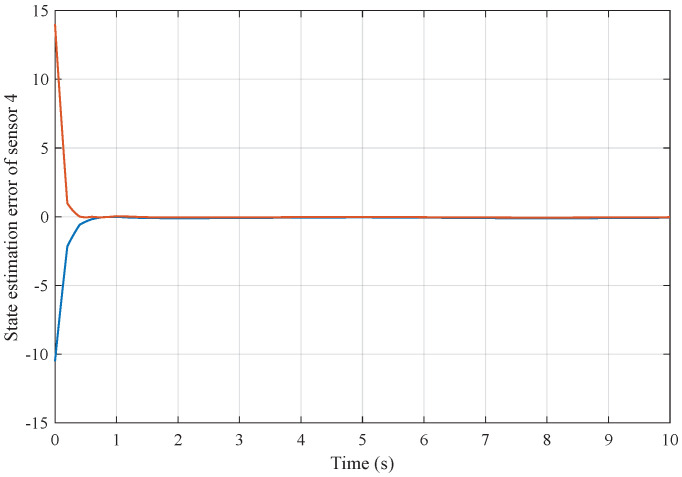
State estimation error of sensor node 4 with 4 sensors.

**Figure 6:**
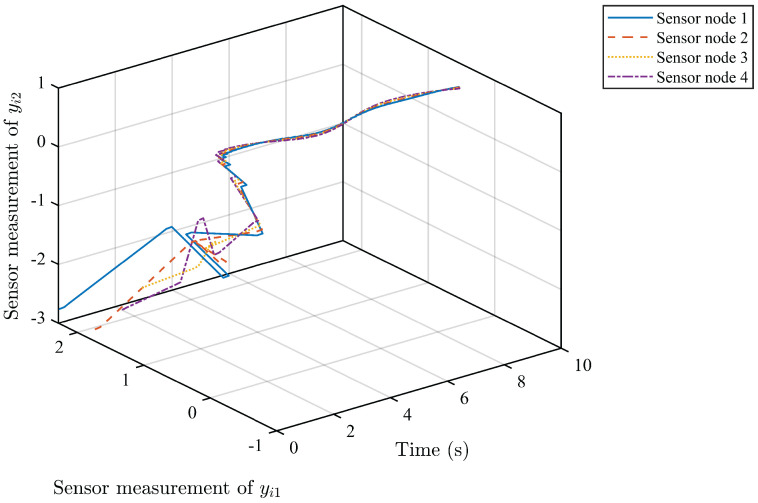
Sensor network output measurement with nonlinearities.

*Based on the above simulation parameters, the state estimation errors of each sensor node with random initial conditions can be seen in Figure 2, Figure 3, Figure 4 and Figure 5, respectively. Meanwhile, the nonlinear measurement output of each sensor node is depicted in Figure 6, respectively. It can be found that although there were certain effects of parameter uncertainties and dynamic nonlinearities, all the sensors could estimate the true states of the plant system well with desired passivity performance.*

**Figure 7:**
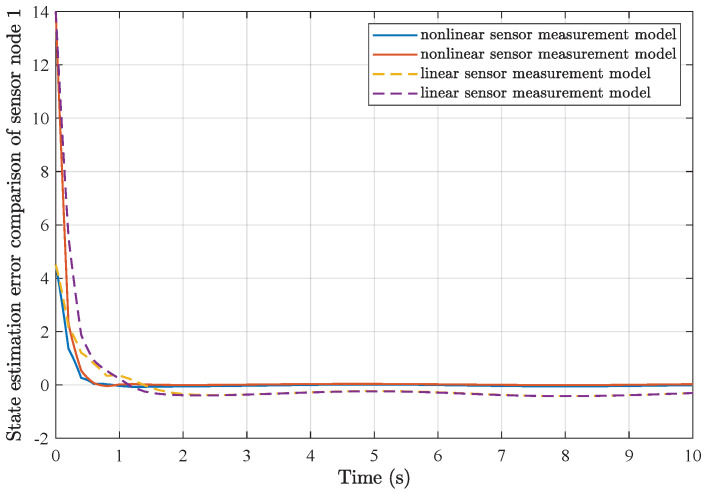
State estimation error comparison of sensor node 1.

**Figure 8:**
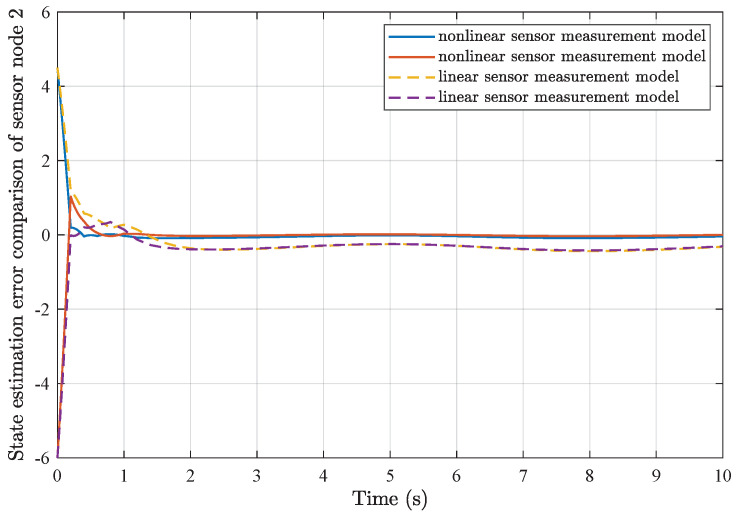
State estimation error comparison of sensor node 2.

**Figure 9:**
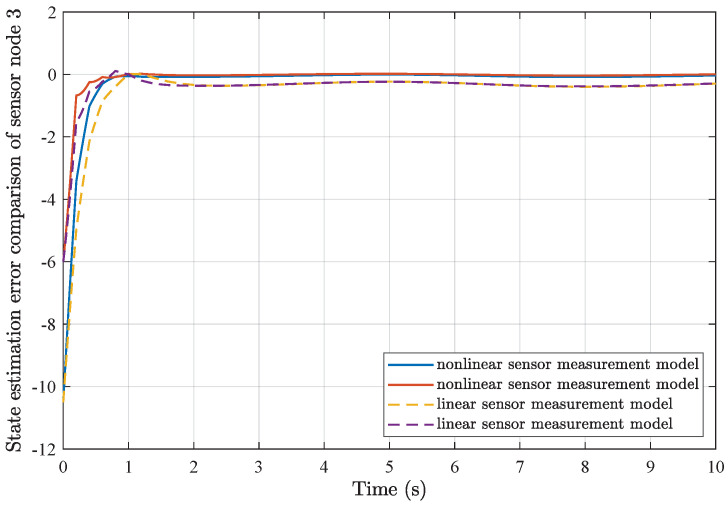
State estimation error comparison of sensor node 3.

**Figure 10:**
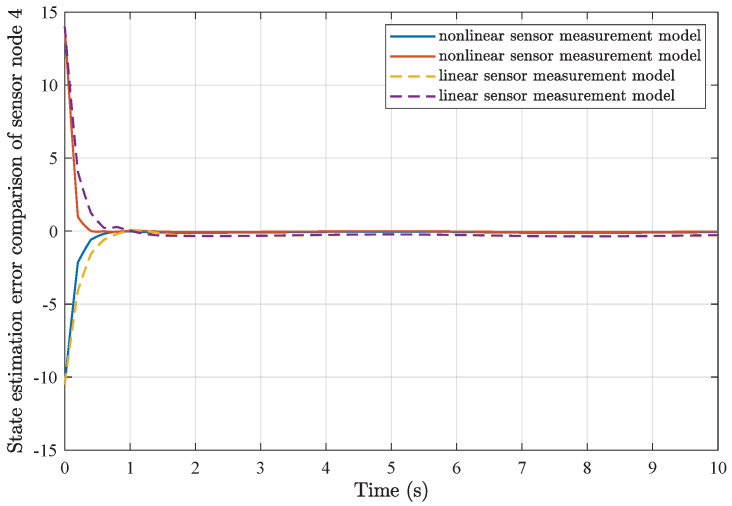
State estimation error comparison of sensor node 4.

*Furthermore, comparisons of simulation results from the utilization of a common linear sensor measurement model and our proposed nonlinear sensor measurement model are depicted in Figure 7, Figure 8, Figure 9 and Figure 10. One can find that the adoption of the nonlinear sensor measurement model could lead to more applicable results with better convergence state estimation dynamics and fewer state estimation errors, such that the advantages of our proposed nonlinear sensor measurement model could be demonstrated accordingly.*

**Figure 11:**
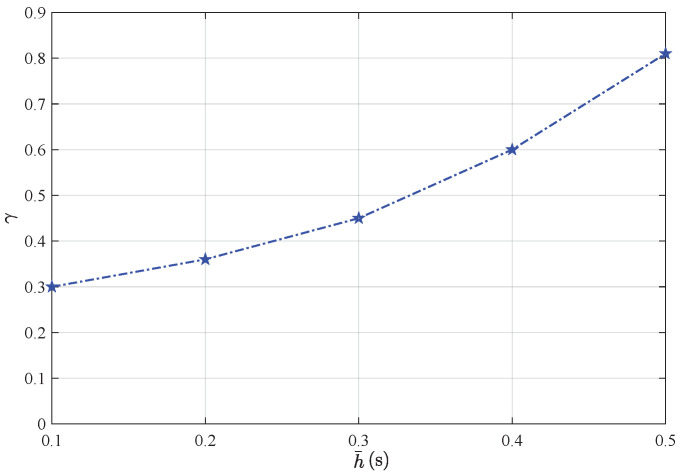
Minimum values of γ with sensor sampling period $\bar{h}$.

*More precisely, the corresponding minimum values of γ with the value of the sampling period $\bar{h}$ are shown in Figure 11. Hence, the correctness and robustness of our designed distributed state estimators can be firmly validated.*

**Example** **2.** *Another simulation example with more sensors is also presented to verify the effectiveness of our developed results.*

*In this example, a group of six sensors was constructed to work collectively for the system in Example 1 according to the topology*

$$A=\left[\begin{array}{cccccc} 1 & 1 & 0 & 0 & 0 & 0\\ 0 & 1 & 1 & 0 & 0 & 0\\ 1 & 0 & 1 & 0 & 0 & 0\\ 0 & 1 & 0 & 1 & 0 & 0\\ 0 & 1 & 0 & 0 & 1 & 0\\ 1 & 0 & 0 & 0 & 0 & 1 \end{array}\right],$$

*where the sensor parameters were set by*

$$\begin{array}{cc} \; & C_{1}=\left[\begin{array}{cc} 1.15 & 0\\ 0 & 1.15 \end{array}\right],\;C_{2}=\left[\begin{array}{cc} 1.1 & 0\\ 0 & 0.95 \end{array}\right],\\ \; & C_{3}=\left[\begin{array}{cc} 0.95 & 0\\ 0 & 0.95 \end{array}\right],\;C_{4}=\left[\begin{array}{cc} 0.9 & 0\\ 0 & 1 \end{array}\right],\\ \; & C_{5}=\left[\begin{array}{cc} 1.10 & 0\\ 0 & 1.10 \end{array}\right],\;C_{6}=\left[\begin{array}{cc} 0.85 & 0\\ 0 & 0.85 \end{array}\right], \end{array}$$

*and*

$$\begin{array}{cc} D_{1} & =\left[\begin{array}{c} 0.1\\ 0.2 \end{array}\right],\;D_{2}=\left[\begin{array}{c} 0.2\\ 0.2 \end{array}\right],\\ D_{3} & =\left[\begin{array}{c} 0.3\\ 0.4 \end{array}\right],\;D_{4}=\left[\begin{array}{c} 0.3\\ 0.2 \end{array}\right],\\ D_{5} & =\left[\begin{array}{c} 0.2\\ 0.3 \end{array}\right],\;D_{6}=\left[\begin{array}{c} 0.1\\ 0.1 \end{array}\right], \end{array}$$

*and the time-varying sampling periods of the sensors were assumed to be h=0.2 s and h=0.25 s.*

*For the passivity performance, it was assumed that γ=2 and the desired state estimator gains $K_{ij}$ were calculated by solving the LMIs in Theorem 2 with the following feasible solutions:*

$$\begin{array}{cc} K_{11} & =\left[\begin{array}{cc} -0.1226 & 0.0462\\ 0.0385 & 0.0386 \end{array}\right],\\ K_{12} & =\left[\begin{array}{cc} 0.0673 & 0.0675\\ 0.0385 & 0.0386 \end{array}\right],\\ K_{22} & =\left[\begin{array}{cc} -0.0922 & 0.0638\\ 0.0372 & -0.1251 \end{array}\right],\\ K_{23} & =\left[\begin{array}{cc} 0.0822 & 0.0822\\ 0.0488 & 0.0488 \end{array}\right],\\ K_{31} & =\left[\begin{array}{cc} 0.0409 & 0.0415\\ 0.0243 & 0.0246 \end{array}\right],\\ K_{33} & =\left[\begin{array}{cc} -0.0514 & 0.0906\\ 0.0544 & -0.0940 \end{array}\right],\\ K_{42} & =\left[\begin{array}{cc} 0.0574 & 0.0576\\ 0.0341 & 0.0342 \end{array}\right],\\ K_{44} & =\left[\begin{array}{cc} -0.0257 & 0.1012\\ 0.0622 & -0.0708 \end{array}\right],\\ K_{52} & =\left[\begin{array}{cc} 0.0747 & 0.0749\\ 0.0426 & 0.0426 \end{array}\right],\\ K_{55} & =\left[\begin{array}{cc} -0.1504 & 0.0479\\ 0.0279 & -0.1784 \end{array}\right], \end{array}$$

$$\begin{array}{cc} K_{61} & =\left[\begin{array}{cc} 0.0485 & 0.0491\\ 0.0263 & 0.0266 \end{array}\right],\\ K_{66} & =\left[\begin{array}{cc} -0.0948 & 0.0293\\ 0.0160 & -0.1129 \end{array}\right]. \end{array}$$

**Figure 12:**
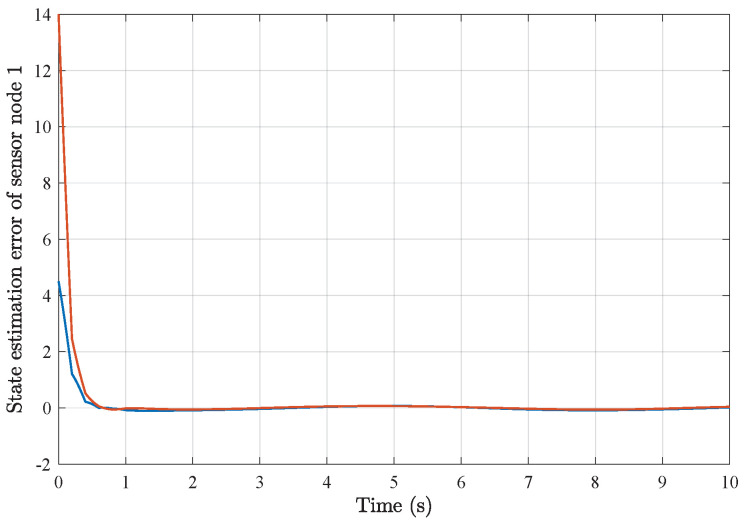
State estimation error of sensor node 1 with 6 sensors.

**Figure 13:**
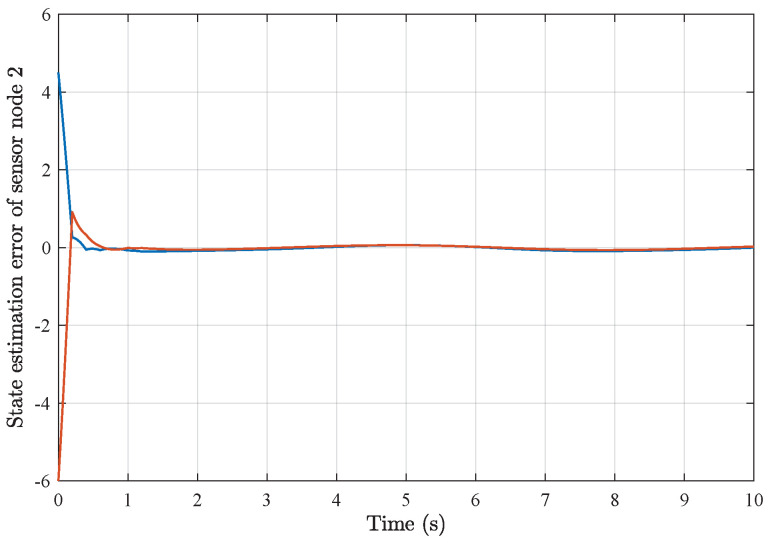
State estimation error of sensor node 2 with 6 sensors.

**Figure 14:**
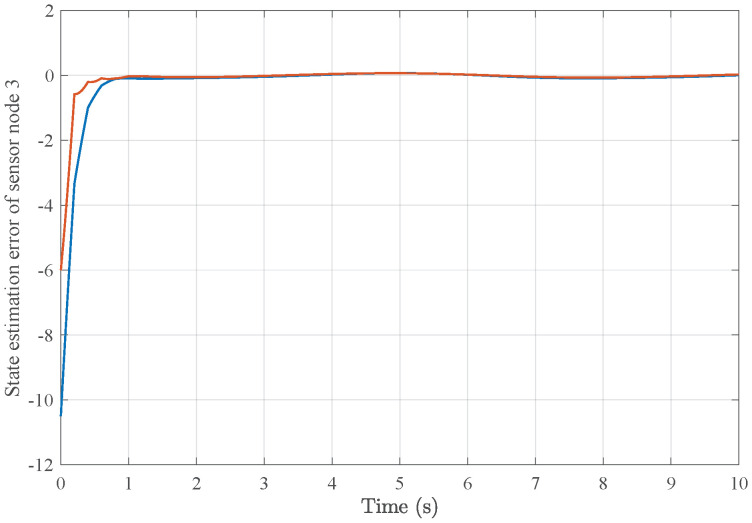
State estimation error of sensor node 3 with 6 sensors.

**Figure 15:**
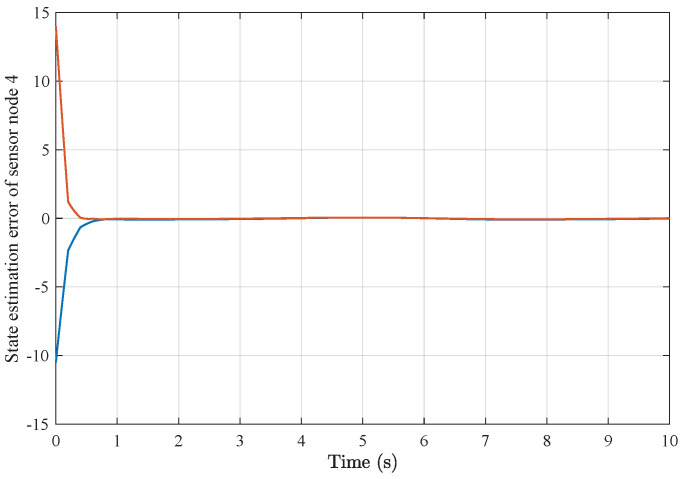
State estimation error of sensor node 4 with 6 sensors.

**Figure 16:**
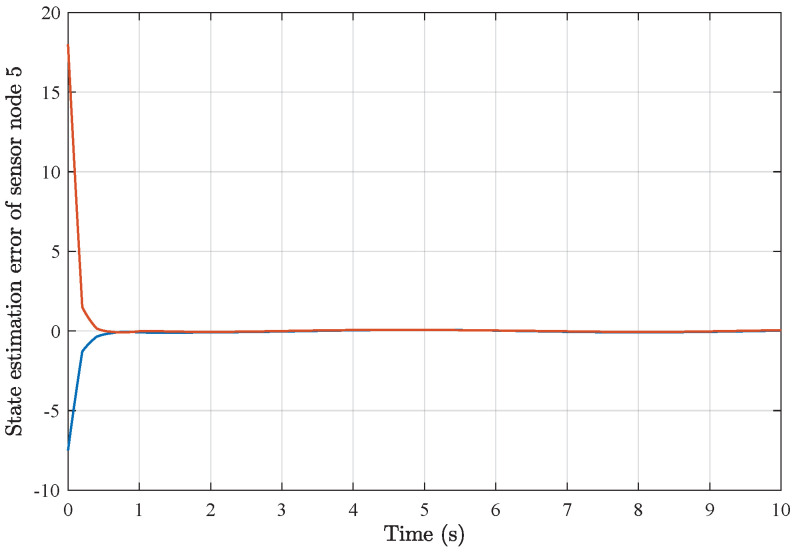
State estimation error of sensor node 5 with 6 sensors.

**Figure 17:**
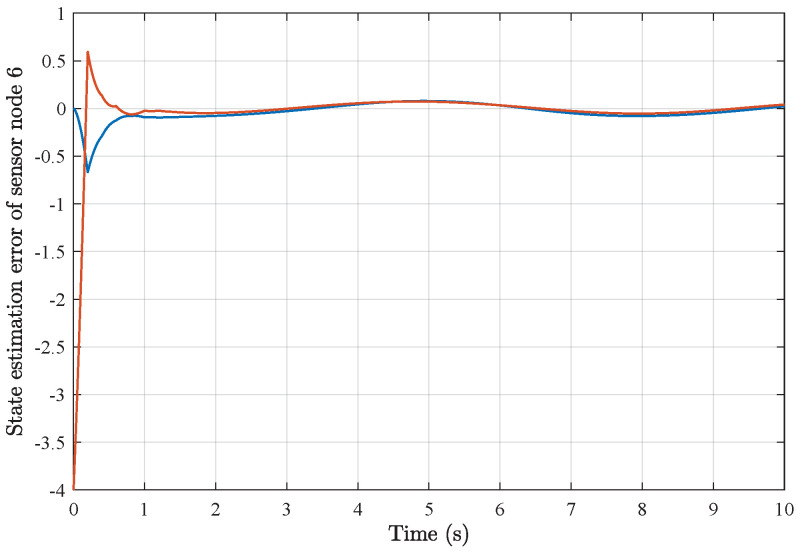
State estimation error of sensor node 6 with 6 sensors.

*As a result of applying the above simulation parameters, the state estimation errors of each sensor node with random initial conditions can be seen in Figure 12, Figure 13, Figure 14, Figure 15, Figure 16 and Figure 17, respectively. These simulation results also confirmed the applicability and generality of the distributed state estimation technique presented in this paper.*

## 5. Conclusions

In our work, the distributed state estimation for a nonlinear system with parameter uncertainties and external disturbances is addressed on the basis of sensor networks. Moreover, the sensor nonlinearity for each sensor measurement is considered and the passivity performance index is adopted to deal with disturbance attenuation. An LMI-based convex optimization method is proposed for the distributed non-fragile state estimator gain design, such that the state estimation error stability and the guaranteed passivity performance can be ensured accordingly. In the end, two simulation studies are provided for verifying the effectiveness of the proposed design methodology. Our future attention will be devoted to more challenging cases of complex sensor nonlinearities as well as those of sensor uncertainties.

## Data Availability

All the data is available and within the paper.
